# Reservoir Equilibrium Development Method by Combined Conformance Control of Polymer/Gel-Dispersed Fluids

**DOI:** 10.3390/gels12060543

**Published:** 2026-06-17

**Authors:** Xin Chen, Jiayi Zhu, Yiqiang Li, Zheyu Liu, Jianbin Liu, Houfeng He, Shun Liu

**Affiliations:** 1 College of Petroleum Engineering, Xi’an Shiyou University, Xi’an 710065, China; 2 College of Petroleum Engineering, China University of Petroleum Beijing, Beijing 102249, China

**Keywords:** elastic-dispersed fluids, conformance control, equilibrium production, slug combination, switching time, EOR

## Abstract

Reservoir conformance control is a necessary production measure in the oil field, which significantly impacts the efficiency of enhanced oil recovery (EOR). Polymers, hydrophobic associating polymers (HAPs), polymer microgels (MGs), and preformed particle gel (PPG) are typical polymer/gel dispersion fluids that are widely used as conformance control agents. Currently, there is still no combined conformance control method to realize the equilibrium production of the reservoir. This paper first evaluates the reservoir adaptability of polymers, HAPs, and MGs by the three-parallel core displacement experiments. Then, the displacement equilibrium factor (DEF) was established by comprehensively considering the profile improvement, oil increment, and oil recovery to optimize the fluid switching time. Based on the above oil displacement experiments, a scatter plot of the DEF with respect to the ultimate recovery of each layer can be plotted, which has an inflection point when the DEF is 45%. When the DEF is lower than 45%, the difference in the oil displacement effect of each layer is enhanced. Therefore, the best time to switch the injection fluid is when the DEF is reduced to 45%. Finally, based on the above results, a graph guiding the combined conformance control method under different reservoir variation coefficients and reservoir median permeability was established, and an equilibrium production method for heterogeneous reservoirs was developed. The five-parallel core flooding experiments with the DEF < 45% as the switching guidance can increase the oil recovery by 17.79% based on association polymer flooding, which is 9.68% higher than that of the conventional conformance control method. This paper can provide theoretical and experimental support for the optimal design of conformance control in oilfields.

## 1. Introduction

Unconventional oil and gas exploration is the mainstream direction of the petroleum industry [1,2,3], but conventional high water cut oilfields still account for the majority of production. Reservoir heterogeneity will lead to the channeling of injected fluid and significantly reduce oil recovery [4,5,6]. Achieving a more uniform production of heterogeneous reservoirs is a key issue faced by laboratory research and field applications [7,8]. Conformance control is an effective technique for balancing water absorption across the multiple layers of a reservoir. With the advancement of theory and synthesis methods, there are numerous conformance control agents [9,10,11,12,13], but two key problems still exist in their actual application process. First is the design of the multi-agent combination mode according to the characteristics of the reservoir, and second is the evaluation of the equilibrium production effect of the reservoir [14].

Polymer flooding can effectively improve the mobility ratio and expand the swept volume, but the profile inversion phenomenon in the late stage of polymer injection is inevitable [15,16]. Gussenov et al. [17] conducted oil displacement experiments after polymer gellan treatment, which increased oil recovery by 9% under relatively unfavorable permeability ratios between the layers (1:2.5). This result proves that the polymer is capable of self-selective placement in high-permeability water-flooded channels. Moreover, researchers have gradually realized that the effect of a single slug on improving the reservoir profile is limited. They found the EOR effects ranked as follows: different polymer slug combinations > same polymer with different viscosity slug combinations > single slug displacement. Zhu et al. [18] established the mobility control theory through the 2D sand-packing model flooding experiments, and proposed a polymer combination injection method with stepwise viscosity reduction. The stepwise viscosity reduction method uses high-viscosity polymer slugs to block the dominant channels, and subsequent low-viscosity polymer slugs entering unswept areas and displacing crude oil. However, polymer solutions are difficult to meet the needs of conformance control at the strongly heterogeneous reservoirs, so the slug combination of polymers and plugging agents has attracted attention [19,20,21]. Saez et al. [22] designed a multi-slug gel injection method to ensure the gel could plug high-permeability layers in a wider range. Alhuraishawy et al. [23] pointed out that compounding PPG with different particle sizes can obtain better conformance control effects. Qiu et al. [24] reported a case of five PPG slug displacements in an oil field (169 t of PPG was injected within 10 months), which increased oil production by 29,600 t and reduced the water cut by 2.2%.

The above studies have clarified that the conformance control method based on slug combination can further expand the swept volume, but there are few studies on the switching timing of each slug. Effective switching between different slugs can avoid waste caused by ineffective circulation of the chemical system and improve economic efficiency. Currently, the switching timing of different slugs is mostly determined by fixing the injection volume of the slugs based on limited experiments [25,26], unable to form a general method. Wu et al. [27] pointed out that injecting high-concentration polymers in advance is beneficial for the subsequent displacement of the remaining oil in the low-permeability layer by low-molecular-weight and low-concentration polymer slugs. However, the results of microfluidic displacement experiments by Liu et al. [28] show that the timing of switching to an S/P slug after water flooding should not be too early or too late. In addition, although the relative permeability curve can be used to quantitatively guide the transfer timing of chemical systems such as polymers [18,29], it is not suitable for conformance control agents [30].

The slug combination conformance control method aims at improving reservoir heterogeneity and realizing the equilibrium production of multi-layer reservoirs. The concept of equilibrium production of heterogeneous reservoirs comes from the idea of improving the effective control degree of reservoirs. In recent years, many scholars have defined the displacement equilibrium factor from different angles. Feng et al. [31] used the maximum net present value of oilfield development to evaluate the equilibrium production of reservoirs. That is, equilibrium production is achieved when the ratio of cumulative oil production in each injection–production direction is equal to the ratio of the pore volume. Chang et al. [32] used the same water cut of a single well as the standard to judge the displacement equilibrium. Liu et al. [33] used the Lorenz curve method to judge the equilibrium production effect of each layer of heterogeneous reservoirs. Chen et al. [34] used the minimum water consumption rate as an index to evaluate the equilibrium production of reservoirs. That is, when the injection pore volume of each layer is the same, the reservoir achieves equilibrium production. Qu et al. [35] proposed using the Theil index to evaluate the equilibrium production degree of the reservoirs.

The above equilibrium displacement factor for evaluating the equilibrium production of reservoirs can be divided into two categories. The first category is divided according to the dynamic production of reservoirs. This method takes the balance of some indicators as the evaluation index, such as achieving the same water cut and liquid absorption in each layer. However, the different physical properties of different reservoirs will inevitably lead to differences in fluid flow, and the pursuit of complete equilibrium is theoretically unfeasible. The other is to use mathematical models to characterize the equilibrium production of reservoirs. The Lorenz curve and the Theil index need to be obtained based on reservoir production parameters, and then the differences in production dynamics at different stages can be obtained from the curves. However, the sensitivity of these methods is low, and it is difficult to clarify the dynamic indicators in the production process in a short period. In other words, the Lorenz curve and Theil index are mainly used for effect evaluation, but are difficult to use for effect prediction and real-time adjustment.

To address the lack of effective methods for confirming the timing of slug combinations, this work established an index to evaluate the reservoir equilibrium utilization effect and used it to develop a real-time adjustment method for multi-stage slug combinations. Firstly, the reservoir adaptability of conformance control agents and EOR effects of different slug combinations were clarified through three-parallel core flooding experiments. Then, considering the fractional flow rate, water cut, and oil recovery comprehensively, the DEF was established, and its dynamic change can guide the switching timing of each slug. Finally, the reservoir equilibrium development method guided by the DEF was summarized and its EOR effect was evaluated through five-parallel core displacement experiments.

## 2. Results and Discussion

### 2.1. The Reservoir Adaptability of HAPs and MGs

**Figure 1:**
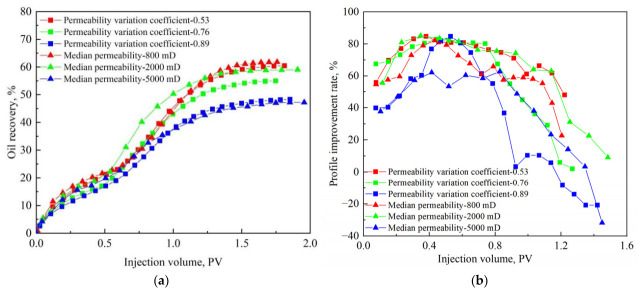
HAP flooding under three permeability variation coefficients and three median permeabilities. (**a**) The oil recovery curves; (**b**) the profile improvement curves.

Figure 1 shows the oil recovery and profile improvement rate curves of HAP flooding with three permeability variation coefficients and three median permeabilities. During the HAP flooding, the reservoir profile improvement rate rises to 80% from 40%, corresponding to the increase in oil recovery, and then the profile improvement rate is significantly reduced in the subsequent water flooding stage. With the increase in the variation coefficient and the median permeability, the final oil recovery reduced significantly. The median permeability of 800 mD has the highest final oil recovery, followed by the median permeability of 2000 mD and the variation coefficient of 0.53. The above results show that the heterogeneity of the reservoir and the absolute permeability directly affect the HAP displacement effect, which can be shown by the profile improvement rate.

When the permeability variation coefficient is 0.89, the profile improvement rate gradually increases from 40% to about 80% and then falls back rapidly while the displacement continues; meanwhile, when the median permeability is 5000 mD, the profile improvement rate also increases slowly and then decreases rapidly. This shows that the increase in the median permeability of the reservoir will reduce the flow resistance of the HAP solution in the high-permeability layer and accelerate the formation of the dominant channel. When the median permeability of the reservoir is constant, the increase in the permeability variation coefficient means a larger difference in the permeability of the high- and low-permeability layers, so the injection pressure required for the fluid to enter the medium- and low-permeability layers will increase. However, the increase in the injection–production pressure difference will also lead to the breakthrough of the HAP solution in the high-permeability layer and form a flow channel.

**Figure 2:**
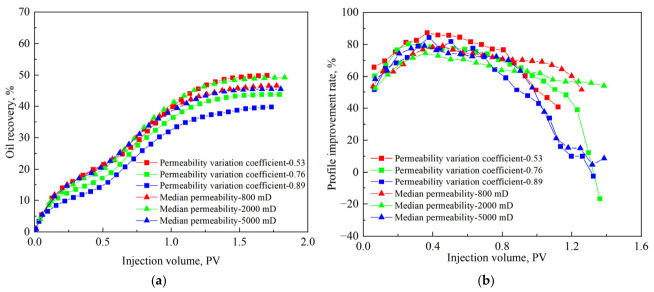
MG flooding under three permeability variation coefficients and three median permeabilities. (**a**) The oil recovery curves; (**b**) the profile improvement curves.

Similarly, Figure 2 shows the oil recovery curves and profile improvement rate curves of MGs under three permeability variation coefficients and three median permeabilities. It can be found that the change tendency is consistent with the HAP flooding. With the increase in reservoir heterogeneity and median permeability, the final oil recovery decreases and the profile improvement effect becomes worse. However, compared with the results of HAP flooding, it can be found that the profile improvement rate of the MG flooding stage is higher than that of the HAP flooding stage, and the decline of the profile improvement rate in the subsequent water displacement stage is low. This shows that MGs have stronger plugging strength than HAPs and the effective action time is longer. Although the profile improvement effect of MG flooding is better, its oil recovery is lower than that of HAP flooding. This is mainly because MGs have a good plugging effect, but do not have viscosity-increasing properties. Although more subsequent fluid can enter the medium- and low-permeability layers, its oil displacement effect is obviously worse than the high-viscosity HAP solution, so the final oil recovery is low.

The key parameters of the oil recovery of the above 12 experiments are summarized in Table 1. The reservoir adaptability of HAPs and MGs can be analyzed from three parameters. (1) EOR effect. For the HAP solution, when the reservoir variation coefficient is 0.53 and 0.76, EOR is basically consistent at about 37%. When the variation coefficient increases to 0.89, EOR decreases by about 5%. Similarly, when the reservoir median permeability is 800 mD and 2000 mD, the EOR of HAPs is approximately 40%. When the median permeability increases to 5000 mD, EOR decreases by about 10%. For the MG solution, in the three reservoir variation coefficients and three median-permeability experiments, its EOR is stable at 26~30%, and is significantly lower than that of HAPs. (2) Final oil recovery. Similarly to EOR, the final oil recovery of HAP flooding decreases with the increase in reservoir variation coefficient and median permeability, and decreases significantly when the variation coefficient reaches 0.89 and the median permeability reaches 5000 mD. The final oil recovery curves of MG flooding are relatively stable, and are generally smaller than that of HAPs. (3) Average profile improvement rate. The average profile improvement rate still shows a trend of decreasing with the increase in reservoir variation coefficient and median permeability. When the reservoir variation coefficient is 0.53 and 0.76, and the median permeability is 800 mD and 2000 mD, the average profile improvement rates of HAPs and MGs are basically the same, indicating that the two have similar profile improvement capabilities. However, when the reservoir variation coefficient increases to 0.89, and the median permeability increases to 5000 mD, the average profile improvement rate of HAPs is significantly lower than that of MGs. At this time, the ability of HAPs to improve the profile deteriorates, and the reservoir adaptability is weakened.

EOR results show that HAPs can achieve good profile improvement and EOR effects under the above six core combinations. However, combined with the final oil recovery and water flooding recovery, it can be found that the increase in reservoir heterogeneity and median permeability will reduce the displacement effect of HAPs, indicating that their ability to improve the reservoir profile has a reservoir application limitation. In contrast, MGs have little change in EOR and final oil recovery with the change in reservoir heterogeneity and median permeability, indicating that they have better profile improvement capabilities. This is also easy to understand. As a solid–liquid dispersion system, the main EOR mechanism of the MG solution is “plugging”, so its oil recovery is inevitably lower than HAPs. Finally, combined with the profile improvement rate and oil recovery, it can be clearly seen that HAPs are suitable for reservoirs with a reservoir variation coefficient less than 0.76 and a median permeability less than 2000 mD; MGs are suitable for reservoirs with a variation coefficient greater than 0.76 and a median permeability above 2000 mD; and when the median permeability of the reservoir increases to more than 10,000 mD, the PPG solution needs to be injected for conformance control, as shown in Table 2.

### 2.2. Slug Combination

**Figure 3:**
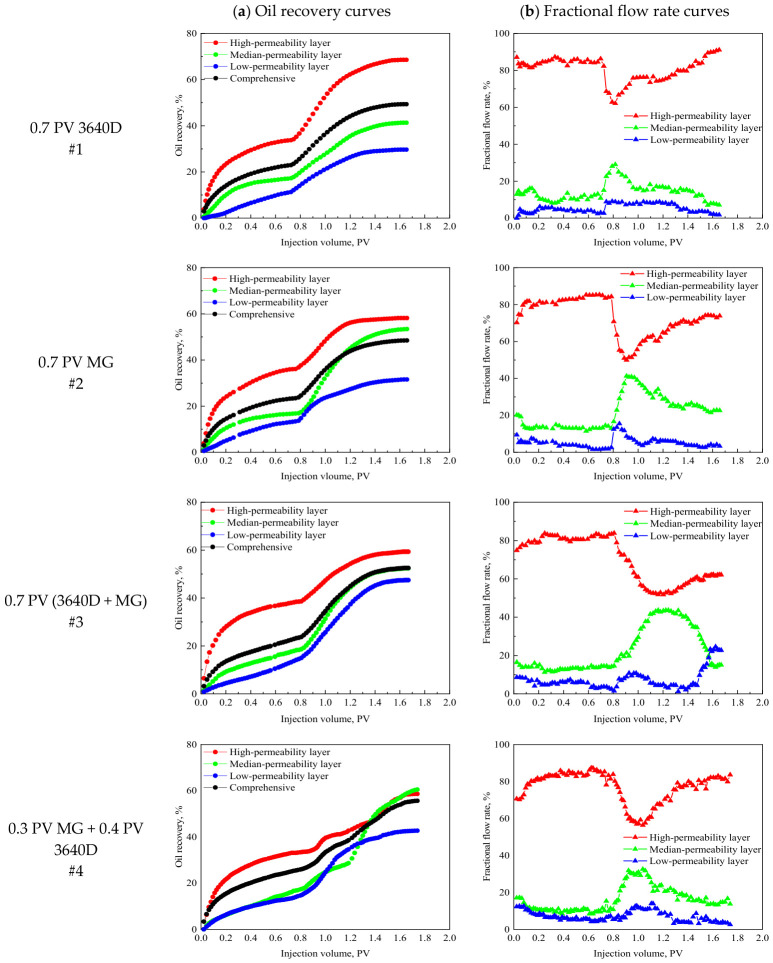

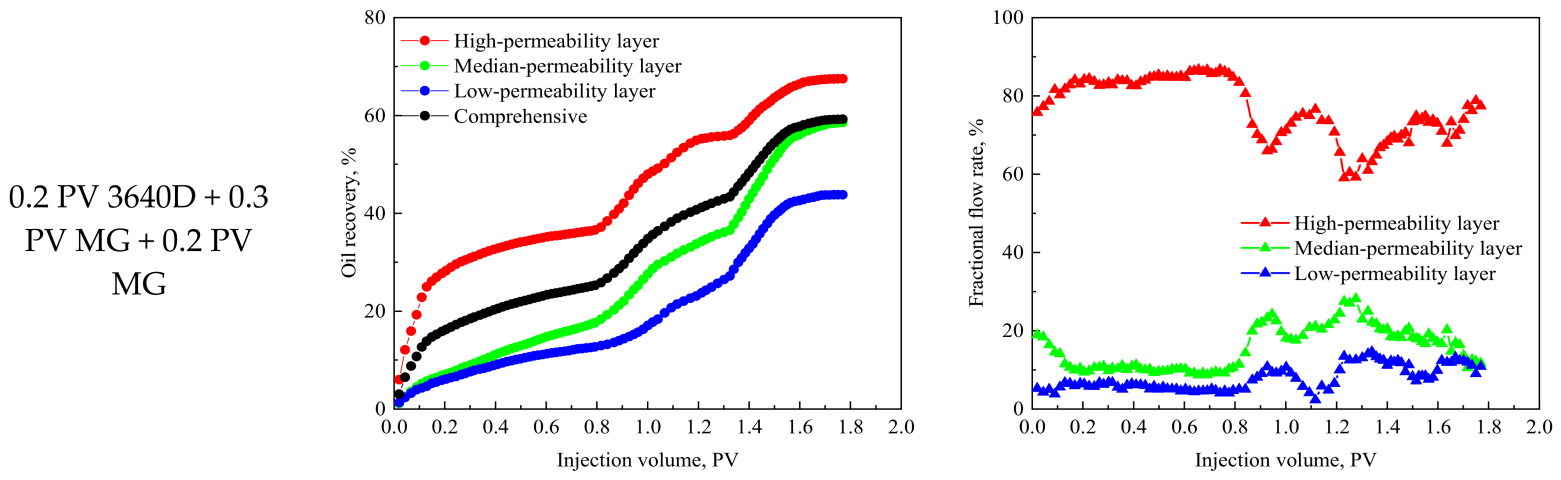
The displacement results of the single slug and slug combination conformance control method. (**a**) Oil recovery curves, (**b**) fractional flow rate curves.

The oil recovery and fractional flow rate curves in Figure 3 show that HPAM flooding mainly produces the middle- and high-permeability layers (In a-1, the blue line is lower and in b-1, the red line is higher). In contrast, the oil recovery of the middle-permeability layer increases rapidly after the injection of MGs, even exceeding that of the high-permeability layer (In a-2 and b-2, the green line is higher and the blue line is the same as a-1). Compared with MGs, the continuous injection of HPAM results in the rapid rise in fractional flow rate of the high-permeability layer, and it will exceed the value at the end of water flooding (The red lines in b-1 and b-2). MGs can effectively reduce the fractional flow rate of high-permeability layers and maintain a good plugging effect in subsequent water flooding. The purpose of the slug combination conformance control method is to delay the re-increase process of the fractional flow rate in the high-permeability layer.

Figure 3 shows that the EOR effect of single-slug flooding is the worst (a-1 and a-2), the EOR effect of the MG and HPAM composite system is enhanced (a-3), and the multi-slug of HPAM and MGs has the best EOR effect (a-4). The EOR of each layer and the comprehensive recovery of each stage in the above five experiments are summarized in Table 3.

MGs can enter and effectively plug high-permeability layers, but it is difficult to greatly displace the oil of high-permeability layers like polymers, leading to the worst EOR effect (only 24.69%); HPAM has the largest EOR of the high-permeability layer (34.49%), but the comprehensive EOR is only higher than that of MG flooding (only 26.37%). Although HPAM can increase the flow resistance of the high-permeability layer, the strength of the profile improvement is weaker than that of MGs. Therefore, the EOR effect of the median- and low-permeability layers is limited, resulting in a low comprehensive EOR; MG and HPAM composite systems do not give full play to the advantages of both. MGs plug the high-permeability layer first after the injection, resulting in lower oil recovery. HPAM can carry more MGs into the medium-permeability layer, resulting in lower oil recovery for the medium-permeability layer (33.2%). However, the compound system produced more oil of low-permeability layers, so its comprehensive EOR (27.99%) is higher than that of their separate injection.

The two-slug displacement of 0.3 PV MG + 0.4 PV HPAM can play to the advantages of the two agents. MGs first plug the high-permeability layer, and the subsequent HPAM mainly enters the median- and low-permeability layers. Although this scheme achieves more EOR in middle- (43.23%) and low-permeability (28.39%) layers, the insufficient production of high-permeability layers (25.27%) results in only a 1.76% increase in EOR compared with experiment 3; the three-slug flooding of 0.2 PV HPAM + 0.3 PV MG + 0.2 PV HPAM can compensate for the above shortcomings. The 1st HPAM slug can fully displace the high-permeability layer (30.31%), then the following MG slug can effectively plug the high-permeability layer. The 2nd HPAM slug is mainly used to displace middle- and low-permeability layers, greatly improving oil recovery. Although the EOR of each layer is not the highest among the five experiments, the comprehensive EOR is the best at 33.37%, realizing the equilibrium production of each layer.

Therefore, the displacement strategy to realize the equilibrium production of heterogeneous reservoirs can be summarized as follows: firstly, fully displace the remaining oil in the high-permeability layer; secondly, plug the high-permeability layer, and then fully produce the sub-high-permeability layer.

### 2.3. Verification of DEF

**Figure 4:**
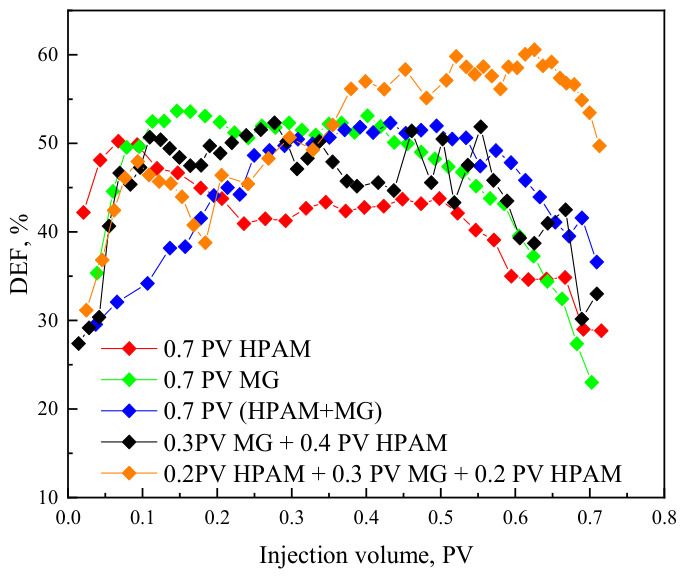
Dynamic change curves of DEF in 5 displacement experiments.

The DEF is defined to calculate the displacement equilibrium degree of the five displacement experiments in Table 8. Here, only the process of injecting 0.7 PV chemical solution is calculated, and the results are shown in Figure 4.

The DEF curve generally rises at the initial injection stage, then remains stable or decreases slowly, and finally decreases rapidly. From the definition of the DEF, it can be seen that it is affected by three parameters, and the main control parameters are different in different stages. The DEF at the initial injection stage is mainly controlled by the profile improvement rate (*λ*), and it begins to decrease slowly after the profile inversion occurs. This can explain that the DEF decline inflection point of HPAM flooding is earlier than that of MG flooding because the profile improvement rate of MGs is always higher. When the composite system of HPAM and MGs is injected, the DEF increases slowly, but the stable time is longer. This is mainly because the strong carrying capacity of HPAM increases the liquid absorption of the high-permeability layer at the initial injection stage, delaying the improvement of the profile. Meanwhile, MGs are also carried into the median-permeability layer, increasing the liquid absorption of the low-permeability layer at the later injection stage; the stability and decrease in the DEF in the middle and late injection stages are mainly controlled by the oil cut rise rate (*ξ*). In the middle injection stage, the middle- and low-permeability layers become the main target of EOR, the oil cut rate increases, and the DEF is generally stable. In the later injection stage, the water cut of the median- and low-permeability layers rises rapidly, and the oil cut rise rate decreases rapidly, resulting in an overall decline in the DEF.

Meanwhile, Figure 4 shows that the DEF will be significantly reduced in the late injection stage of a single slug, which means that the improvement effect of the profile and the development effect of the median- and low-permeability layers will worsen. The slug combination conformance control method can effectively alleviate the decline of the DEF in the later injection stage of the previous slug, which is also the performance of equilibrium production of reservoirs. Therefore, the combination timing of each slug can be determined according to the dynamic DEF curve in the displacement process to avoid invalid or low-efficiency circulation of the injection system.

### 2.4. Timing of Slug Combinations

#### 2.4.1. Oil Displacement Efficiency

**Figure 5:**
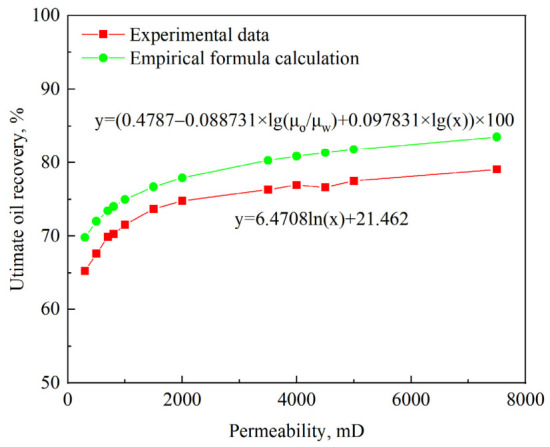
Variation curves of polymer flooding oil displacement efficiency in different permeability cores.

Figure 5 shows the HPAM flooding efficiency curve of cores with effective permeability ranging from 300 mD to 7500 mD. It can be found that with the increase in the effective permeability of the core, the oil displacement efficiency first increases obviously and then tends to be stable. The overall change is a logarithmic function curve. Yu et al. [36], based on the water flooding data of 25 oilfields in China, regress the empirical formula of water flooding efficiency on oil–water viscosity ratio and effective permeability (Equation (1)).

(1) $$E_{D}=0.4787-0.08873\mathrm{lg}\mu_{R}+0.09783\mathrm{lg}K$$

where *E_D_* is the ultimate oil displacement efficiency, %; *μ_R_* is the viscosity ratio of the oil and water phase; *K* is the effective permeability, mD.

In this paper, the viscosity of the polymer solution is used instead of the viscosity of the water to calculate the curve of the ultimate recovery factor with the permeability using Equation (1) and compare it with the experimental value. Figure 5 shows that the HPAM displacement efficiency curve obtained in the experiment has the same trend as the curve calculated by the empirical formula.

#### 2.4.2. Determination of Slug Combination Timing

**Figure 6:**
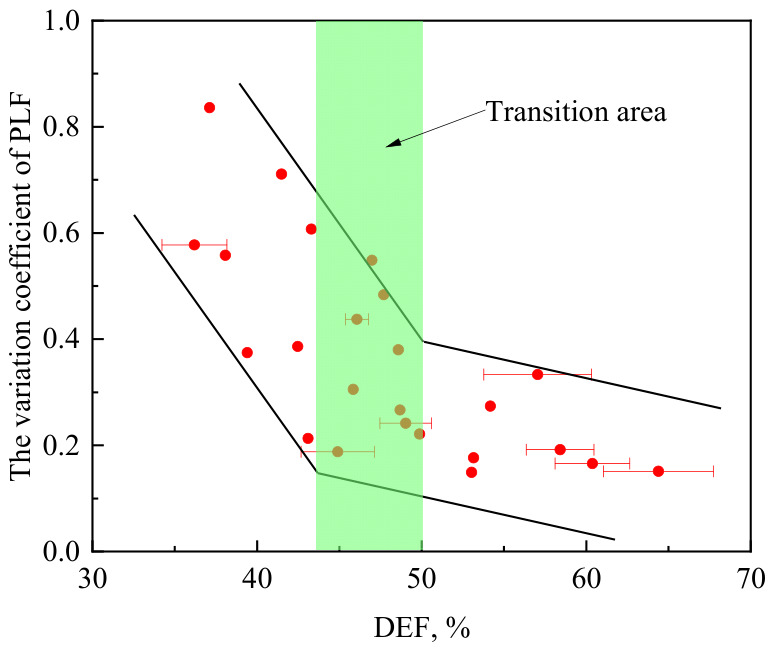
Scatter diagram of DEF and the variation coefficient of PLF.

The scatter diagram of the DEF and the variation coefficient of PLF for the 24 groups of experiments in Table 8 is shown in Figure 6, in which the DEF is the maximum value in each displacement experiment. The overall distribution of the DEF is between 35% and 65%, while the variation coefficient of PLF is between 0.1 and 0.8. When the DEF decreases from 65% to 50%, the variation coefficient of PLF increases slowly. When the DEF continues to decrease, the variation coefficient of PLF increases rapidly. This shows that when the DEF is reduced to a certain range, the overall oil displacement effect of the heterogeneous reservoir will be significantly reduced. At this time, the development mode should be adjusted, that is, the injection slug should be changed. It can be found that when the DEF is 45%, it is the inflection point of the variation coefficient of PLF, which corresponds to the optimal timing for slug switching.

In summary, through the definition of the DEF and the analysis of a large number of experimental data, it can be clarified that when the DEF decreases to 45%, it is the best time for slug conversion of the chemical system. Figure 6 was obtained under different permeability, heterogeneity, and flooding conditions, with error bars confirming the reliability of the experimental data. Therefore, the inflection point at 45% is highly objective. Moreover, this inflection point indicates a rapid decline in the EOR efficiency of the injection agent, serving as a clear and significant indicator. Whether it is an indoor experiment or a field application, the DEF can be calculated in real time based on obtaining the physical properties and fluid production profile of each layer. To enhance the applicability of the DEF, future experiments are needed to investigate the influence patterns of parameters such as crude oil viscosity and injection rate.

### 2.5. Combination Conformance Control Graph and Reservoir Equilibrium Development Method

**Figure 7:**
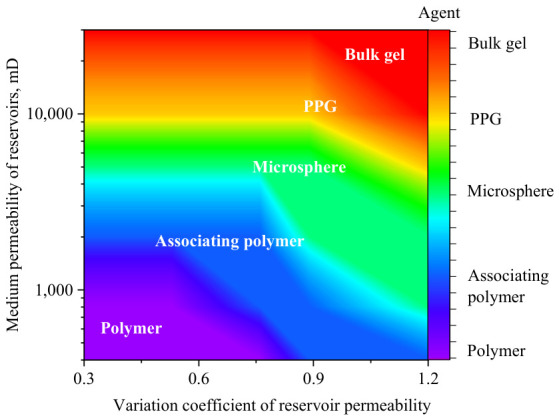
Slug combination conformance control graph.

This paper comprehensively considers the heterogeneity and permeability of the reservoir to establish a slug combination conformance control graph to achieve the reservoir equilibrium development, as shown in Figure 7. The horizontal axis of the graph is the reservoir variation coefficient (represents the reservoir heterogeneity), and the vertical axis is the median permeability of the reservoir (represents the size of the reservoir pore throat). The equilibrium development of water flooding gradually deteriorates from the lower left corner to the upper right corner of the graph, and the difficulty of conformance control also gradually increases. The transition from the purple area to the red area in the graph represents the increase in the plugging strength of the required agent, i.e., polymer, HAPs, MGs, PPG, and gel. Figure 7 is drawn based on the 24 sets of experimental results in Table 8. For a given reservoir (permeability and heterogeneity), the type and intensity of agents required for conformance control can be initially selected based on Figure 7, which greatly reduces the workload of indoor evaluation.

The above results can solve the three main problems throughout the whole displacement process: optimization of the displacement solution, combined injection plan, and slug conversion timing. They can be summarized as a method for the reservoir equilibrium displacement based on a slug combination conformance control method.

(1) Optimize the displacement system. According to the slug combination conformance control graph shown in Figure 7, select the appropriate conformance control agent (such as the shaded part in Figure 7) based on the physical properties of the target reservoir. Then, select the physical parameters of each agent according to the matching plugging model, such as HAP solution concentration, MGs, and PPG particle size, etc.;
(2) Clarify the combination of the displacement agents. The displacement agent combination is designed with the idea of “multiple rounds and multiple slugs, and from weak to strong”. After water flooding, polymer or HAP slug displacement is given priority, and the conformance control agents are followed after the high-permeability layer is fully utilized. Meanwhile, to give full play to the role of “conformance control” and “displacement”, a polymer slug should be conducted after the conformance control slug to enhance the oil recovery effect of the medium- and low-permeability layers;
(3) Dynamically control the timing of the slug combination. The effect of the injected slug can be judged based on the DEF. When the effect of the slug is lower than 45%, the slug replacement is carried out, which can effectively avoid the ineffective and inefficient cycle of a single slug.
(4) Complete plugging of the high-permeability layer. For highly heterogeneous reservoirs, the low-permeability layer still has a high potential for development after the application of the slug combination conformance control. At this time, the gel can be injected to completely plug the high-permeability layer, and the method for the reservoir equilibrium displacement based on the slug combination conformance control method can be carried out again (that is, the above three steps are repeated) to further develop the low-permeability layer.

It is particularly important to note that the combined conformance control method is implemented step by step under the condition that all monitoring data are normal. However, the conformance control system has strong plugging properties, and abnormal pressure rise phenomena are more likely to occur during the injection process. Although the application of the above method requires ensuring the compatibility between the agent and the reservoir, in actual application, a certain limit pressure (i.e., the increase in injection pressure) should be set. Once the pressure exceeds the limit, the agent with better injectivity should be replaced. Conducting the above stimulation process under the condition of limit pressure can avoid excessive particle retention in the reservoir, which may cause reservoir damage.

### 2.6. EOR Effect of Slug Combination Conformance Control Method Under the Guidance of DEF

#### 2.6.1. EOR Effect of Conventional Conformance Control Method with One PPG Slug

**Figure 8:**
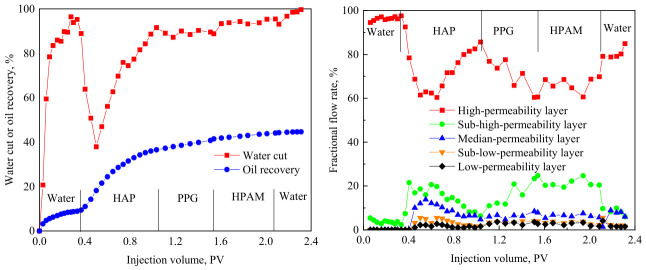
Curves of oil recovery, water cut, and frictional flow rate in conventional conformance control methods.

According to the reservoir equilibrium development method summarized in Section 2.5, two five-parallel displacement experiments were designed to verify the beneficial effects of this method. The curves of oil recovery, water cut, and fractional flow rate of the conventional conformance control method are shown in Figure 8. It can be found that the water cut in the water flooding stage rises rapidly and enters the high water cut stage when the injection volume is about 0.3 PV. The fractional flow rate of the high-permeability layer is stable at about 95%, and only the high-permeability and sub-high-permeability layers have liquid produced. After injecting the HAP solution, the water cut decreased rapidly, and the EOR effect was obvious; meanwhile, the fractional flow rate of the high-permeability layer was significantly reduced, and the liquid was produced in all five layers; after injecting about 0.2 PV of HAP solution, the water cut and fractional flow rate of high-permeability layer began to rise, and the growth rate of oil recovery slowed down. The high-permeability layer became the dominant channel again.

#### 2.6.2. EOR Effect of the Slug Combination Conformance Control Method

**Figure 9:**
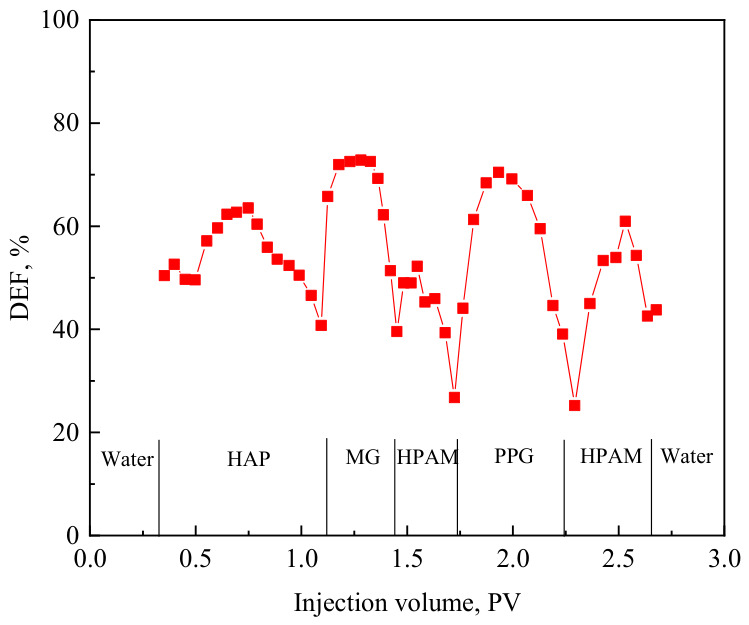
DEF curve of the displacement process of the multi-agent and multi-slug conformance control method.

The switching timing of the slug combination conformance control method is determined by the change in the DEF in the displacement process. According to Equation (8), the DEF curve of the five-pipe parallel displacement process is shown in Figure 9.

It can be seen that after HAP flooding, the DEF first gradually increased, then decreased slowly, and finally decreased rapidly. The DEF decreased to 40.49% when the injection volume reached 0.7 PV, and MG flooding was transferred. During MG flooding, the DEF rose rapidly to more than 70% and remained stable. At this time, the profile improvement rate plays a major control role. When the injection rate of MGs was about 0.25 PV, the DEF decreased rapidly. Currently, the profile improvement rate and the oil cut rise rate jointly determine the change in the DEF. The DEF decreased to 39.32% after injecting 0.35 PV of MG solution, and the HPAM solution was transferred to play the role of oil displacement; after the transfer, the DEF was stable at about 50%. At this time, although the oil cut rise rate increased, the decrease in the profile improvement rate resulted in a small increase in the DEF. The DEF decreased to 26.77% after injecting 0.25 PV of HPAM, and the PPG solution was transferred to further plug the high-permeability layer; after the PPG injection, the DEF climbed to 70% again, which mainly depends on the oil cut rise rate. After PPG was injected, the profile improvement rate increased and remained stable, while the oil recovery rate increased significantly first and then decreased rapidly after reaching the peak. The DEF decreased to 39.08% after injecting 0.5 PV of PPG, and the HPAM solution was transferred again to exert the oil displacement effect after conformance control; during the second HPAM flooding, the DEF rose to about 50%, which also lasted for a short time, but the overall value was higher than that during the first HPAM flooding stage after MG flooding, indicating that the profile improvement effect of PPG is obviously better than that of MGs. The DEF decreased to 43.77% after 0.4 PV of HPAM injection, and it was transferred to subsequent water flooding. Both the profile improvement rate and the oil cut rise rate in the subsequent water flooding stage were greatly reduced, so the DEF was not calculated. Therefore, the slug combination conformance control process is water flooding to a comprehensive water cut of 90% + 0.7 PV HAP + 0.35 PV of MG + 0.25 PV HPAM + 0.5 PV PPG + 0.4 PV HPAM + subsequent water flooding.

**Figure 10:**
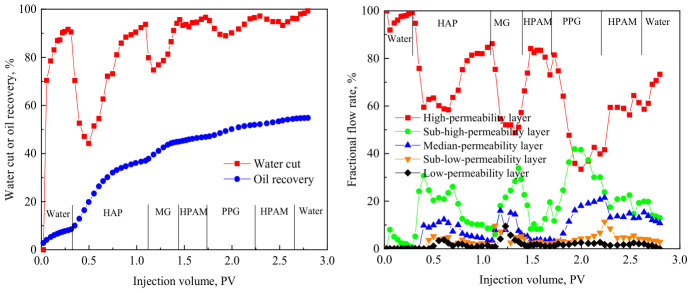
Curves of oil recovery, water cut, and fractional flow rate of the multi-agent and multi-slug conformance control method.

The oil recovery, water cut, and fractional flow rate curves of the slug combination conformance control method are shown in Figure 10. It can be found that the change law of each displacement characteristic parameter in the water flooding stage and the HAP flooding stage is basically consistent with the conventional conformance control method. In the follow-up MG and PPG flooding stages, the comprehensive water cut and the fractional flow rate of the high-permeability layer decreased significantly, showing a funnel shape. The HPAM slug behind the PPG/MG can delay the rising trend of water cuts and produce crude oil from reservoirs with low permeability. The slug combination conformance control method can plug the high-permeability layer after it has been fully produced and play the role of displacement in the lower-permeability layer. The final oil recovery was stable at 54.83%, and the EOR increased to 17.79%, which is relatively 9.69% higher than the conventional method. The application of this method in the Bohai Oilfield has also verified its advanced nature [37].

#### 2.6.3. The Contrast of the EOR Effects

The key oil recovery parameters of the conventional conformance control method and the combination conformance control method were summarized and compared in Table 4. Here, the HAP slug can fully displace the high-permeability layer, which is a main EOR part of the five-pipe parallel displacement. In the follow-up stage of PPG/MG flooding, the effective targets for EOR are mainly median- and low-permeability layers. The combination conformance control method can enhance oil recovery of the median-permeable layer to 31.23%, which is relatively 9.11% higher than the conventional method. This is mainly because the slug combination conformance control method can effectively avoid polluting the median- and low-permeability layers and realize their full production by temporarily plugging the high- and sub-high-permeability layers.

**Figure 11:**
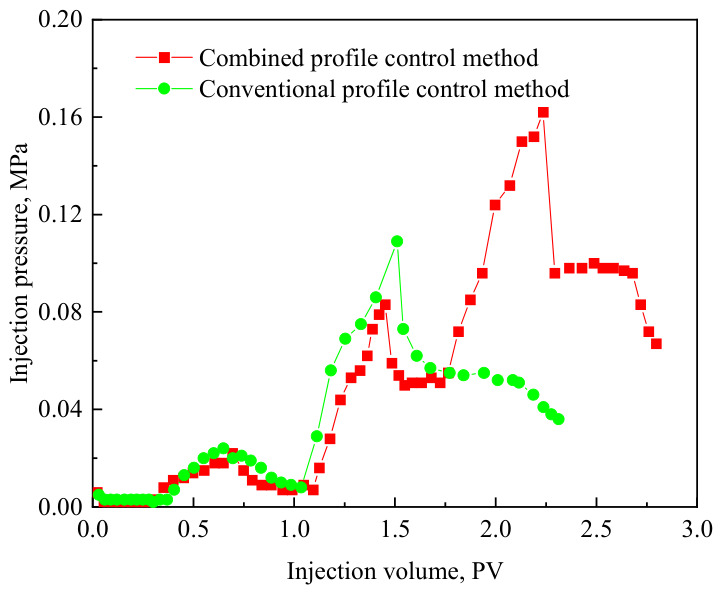
Injection pressure curves of the conventional conformance control method and the slug combination conformance control method.

The advantage of the slug combination conformance control method lies in the full production of the medium-, sub-low-, and low-permeability layers. The production of sub-low-permeability layers and low-permeability layers is reflected in the accumulation of injection pressure. The injection pressure curves of the two methods are shown in Figure 11. The injection pressure will increase significantly after the injection of MGs and PPG. The pressure of the following HPAM slug will drop but remain at a relatively high level. This shows that the multi-slug combination can effectively accumulate the injection pressure and fully produce the lower-permeability layers.

## 3. Conclusions

This paper studied the reservoir adaptability of HPAM, HAPs, and MGs and the combination effect of these conformance control agents. Meanwhile, the DEF was defined to characterize the equilibrium utilization of the reservoir, and the timing of the agent combination was regulated based on this. Finally, a reservoir equilibrium displacement method for heterogeneous reservoirs is established. The specific conclusions are as follows.

(1) HAPs are suitable for reservoirs with a permeability variation coefficient less than 0.76 and a median permeability less than 2000 mD; MGs are suitable for reservoirs with a variation coefficient greater than 0.76 and a median permeability above 2000 mD; and when the median permeability of the reservoir increases to more than 10,000 mD or there are some fractures, PPG solution needs to be injected for conformance control. The slug combination conformance control graph was established and can be used for the agent optimization of the target heterogeneous reservoir.
(2) The EOR effect of the slug combination conformance control method has a better conformance control effect. The idea of the multi-agent combination can be summarized as follows: First, HPAM/HAPs are used to fully displace the oil in the high-permeability layer, and then MGs are used to effectively plug the high-permeability layer to develop the crude oil in the medium- and low-permeability layers.
(3) Considering fractional flow rate, water cut, and oil recovery, the DEF was established to evaluate the conformance control effectiveness. The DEF rises at the beginning after the conformance control agent injection, then remains stable or decreases slowly, and finally decreases rapidly. The change in the DEF proves that the conformance control effect of a single slug decreases significantly in the middle and late injection stages.
(4) The scatter diagram of the DEF and variation coefficient of DFL shows that there is an inflection point when the DEF is about 45%, after which the continuous decrease in the DEF will rapidly increase the difference in the oil recovery of each layer. Therefore, the switching timing of each slug is taken as the DEF lower than 45%.
(5) Five-parallel core flooding experiments show that after HAP flooding, the slug combination conformance control method can increase the oil recovery by 9.69% compared with the conventional conformance control method. This is mainly attributable to the substantial production of medium-, sub-low-, and low-permeability layers.

## 4. Materials and Methods

### 4.1. Materials

**Figure 12:**
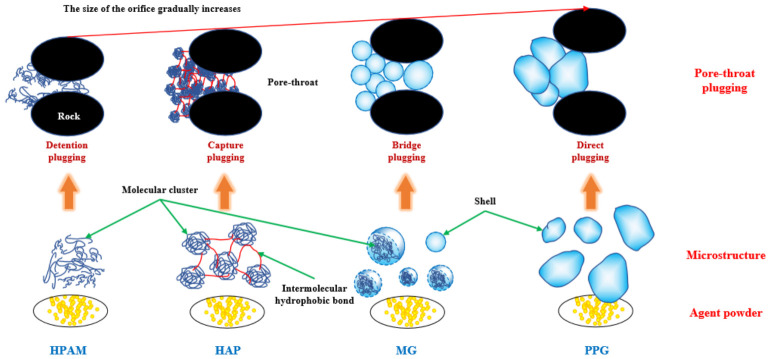
Comparison of the microstructure and blocking mechanism of the four agents.

The simulated oil is a compound of degassed and dehydrated crude oil and kerosene, with a viscosity of 58 cP at 55 °C. Deionized water (DI water) was produced by UPT-I-10T Ultra-pure Water Purifier from Chengdu Youpu Super Pure Technology Co., Ltd., Chengdu, China. The simulated formation water is prepared by adding inorganic salts to DI water in proportion, and the total salinity is 5997 mg/L. The elastic-dispersed fluids include polymer (represented by HPAM), hydrophobic association polymer (represented by HAP), polymer microgels (represented by MGs, median sizes are 8.3 μm and 20.0 μm), and PPG (median size is about 400 μm). The molecular structures of the above four agents range from loose to compact as shown in Figure 12 and their advantages and disadvantages are compared in Table 5. The core work of this paper is to find the optimal agent based on reservoir conditions. HPAM and PPG dry powder are commercial products, and HAPs and MGs are synthesized in the laboratory [3,38]. The above agents are non-toxic, harmless, and environmentally friendly. The MG and PPG solution belong to the solid–liquid dispersion system, and it is necessary to use a stirring piston to prevent particle precipitation.

The cores used in the experiment are artificial square sandstone cores of 4.5 cm × 4.5 cm × 30 cm. According to the experimental requirements, the water permeability ranges from 300 mD to 10,000 mD. The parallel connection of cores with different permeabilities can simulate reservoir heterogeneity. This paper mainly involves the three-parallel core and five-parallel core models. Among them, the three-parallel cores model is used to evaluate the reservoir adaptability of the agents and optimize the system combination mode and conversion timing. By changing the permeability of the three cores, three median permeabilities and three permeability variation coefficients can be obtained. The specific parameters are shown in Table 6.

The five-parallel cores model was used to evaluate the beneficial effects of the slug combination conformance control method based on DEF established in this paper. The parameters of the five-parallel cores model are shown in Table 7.

### 4.2. The Reservoir Adaptability of Elastic-Dispersed Fluids

HAPs have a much higher viscosity than HPAM, while MGs and PPG have solid–liquid dispersion characteristics. So, these agents are suitable for plugging in high-permeability and ultra-high-permeability reservoirs, respectively. The matching relationship between the agents and the high-permeability layer is the key to plugging the channeling layer, achieving conformance control, and expanding the swept volume. Here, the HAP and MG parameters under different core parameters can be designed according to the previous research results [38]. The particle size of PPG is too large, and it is usually necessary to use a thin tube model or a microfluidic model for experimental evaluation [39]. The matching relationship between the particle size and permeability is shown in Table S1 to Table S3, respectively. Based on this, the experimental scheme can be designed as shown in Table 8 (columns A and B).

**Figure 13:**
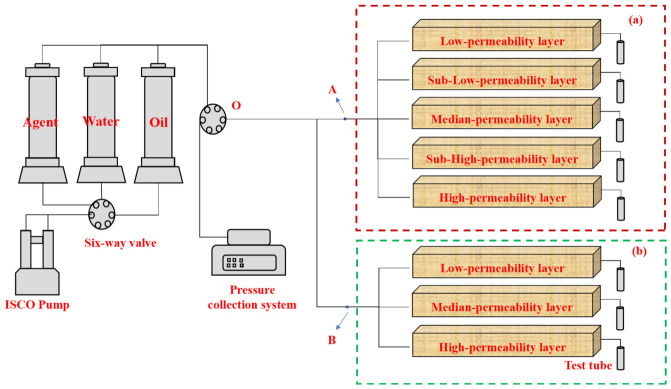
The flow chart of parallel cores displacement. (**a**) Five-parallel cores flooding experiment, (**b**) three-parallel cores flooding experiment.

The specific procedure of displacement experiment is as follows: (1) Vacuum the dried cores for 2 h, then self-absorb saturated water for 4 h to calculate the porosity; (2) Connect the experimental procedure according to Figure 13b (connect point O to point B), and calculate the water permeability based on the Darcy law; (3) Use the ISCO pump to inject crude oil into the water-saturated cores until there is no water produced. The produced water volume represents the saturated oil volume, and the oil saturation rate can be calculated; (4) Put the core in a 55 °C thermostat for 2 days, then carry out a constant-speed oil displacement experiment, and record the fluid production of each layer and the change in injection pressure. In practice, using an internal pressure tap can provide the true pressure inside the core, avoiding misjudgments caused by increased injection pressure due to end-surface blockage. Previous research by the authors has already conducted injectivity and plugging ability of conformance control agent evaluation experiments [39]; therefore, the injection end pressure is used here to represent the pressure difference between the two ends of the core, ensuring that HPAM, HAPs, and MGs all enter the core. The displacement process is: water flooding until the water cut reaches 80%, conformance control solution injection for 0.7 PV, and subsequent water flooding until the water cut reaches 98% to end the experiment. The experiment was carried out in a thermostat with a constant temperature of 55 °C, and the injection rate was 1 mL/min.

### 4.3. Optimization of Elastic-Dispersed Fluids: Slug Combination

Three cores with water permeabilities of about 700 mD, 2000 mD, and 3500 mD were connected in parallel to simulate the reservoir heterogeneity. Flooding of single-slug (HPAM, MG, HPAM, and MG binary solution), two-slug (HPAM and MG combination), and three-slug (HPAM and MG combination) combinations were carried out. The specific experimental schemes are shown in Table 9. The special experimental steps and process are the same as in Section 2.2.

### 4.4. Establishment of DEF

Equilibrium displacement refers to maximizing the production of multi-layered reservoirs while minimizing the interlayer gaps [40,41]. The swept volume of the injected fluid is mainly affected by the dynamic flow resistance of the injected fluid. According to the hydroelectric similarity theory, the Darcy law (Equation (2)) is deformed to obtain the flow resistance of the fluid, as shown in Equation (3). When the physical model or reservoir conditions are constant, the flow resistance is only related to the ratio of viscosity to permeability, that is, the reciprocal of mobility. Therefore, mobility can be used to characterize the displacement equilibrium.

(2) $$Q=\frac{KA\Delta P}{\mu L}\times 100\%$$

(3) $$F_{R}=\frac{\mu L}{KA}$$

(4) $$\frac{A\Delta P}{L}=\frac{\mu_{1}Q_{1}}{K_{1}}=\frac{\mu_{2}Q_{2}}{K_{2}}=\cdots =\frac{\mu_{i}Q_{i}}{K_{i}}$$

where *Q* is the injection velocity, mL/s; *K* is the water permeability, mD; *A* is the section area, cm^2^; Δ*P* is the injection pressure, MPa; *μ* is the viscosity of the injection solution, mPa·s; *L* is the length of the cores, cm; *F_R_* is the flow resistance; Subscripts 1–*i* represent the number of the formation layer.

However, the mobility calculation involves the change in fluid viscosity and reservoir permeability during the displacement process, which is difficult to characterize quantitatively. And by deforming Darcy law again, Equation (4) can be obtained. For the multi-layer reservoir, the right side of Equation (4) is a constant value, and the flow rate of each layer is positively correlated with flow resistance. Therefore, the change in the flow rate of each layer is used to characterize the change in flow resistance during the displacement process.

In addition, DEF is also affected by the water cut and oil recovery of each layer. Three index parameters (profile improvement rate *λ*, oil cut rise rate *ξ*, and oil recovery equilibrium factor *θ*) are defined to describe the fraction flow rate, water cut, and oil recovery of each layer, respectively, as shown in Equations (5)–(7). *λ* is a dimensionless parameter calculated based on the rate of change in production of each layer during different displacement stages in a multi-layered reservoir, and can be used to evaluate changes in water absorption profile. *ξ* is a dimensionless parameter calculated based on the water cut of each layer during different displacement stages in a multi-layered reservoir, and can be used to evaluate changes in oil production. *θ* is a dimensionless recovery rate calculated based on the recovery rate of each layer during different displacement stages in a multi-layered reservoir, and can be used to evaluate differences in recovery rates among layers.

(5) $$\lambda =\frac{Q_{hw}/(Q_{mw}+Q_{lw})-Q_{hp}/(Q_{mp}+Q_{lp})}{Q_{hw}/(Q_{mw}+Q_{lw})}$$

(6) $$\xi =\frac{\left[Q_{m}^{\prime }\times (1-f_{wm}^{\prime })-Q_{m}\times (1-f_{wm})]+[Q_{l}^{\prime }\times (1-f_{wl}^{\prime })-Q_{l}\times (1-f_{wl})\right]}{Q_{l}^{\prime }\times (1-f_{wl}^{\prime })+Q_{m}^{\prime }\times (1-f_{wm}^{\prime })+Q_{h}^{\prime }\times (1-f_{wh}^{\prime })}\times 100\%$$

(7) $$\theta =\frac{\left[(1-\eta_{Lw}/\eta_{Hw})+(1-\eta_{Mw}/\eta_{Hw})]-[(1-\eta_{Lp}/\eta_{Hp})+(1-\eta_{Mp}/\eta_{Hp})\right]}{\left[(1-\eta_{Lw}/\eta_{Hw})+(1-\eta_{Mw}/\eta_{Hw})\right]}\times 100\%$$

where *Q_hw_*, *Q_mw_*, and *Q_lw_* are the fractional flow rate of high-, median-, and low-permeability layers at the end of water flooding, respectively, %; *Q_hp_*, *Q_mp_*, and *Q_lp_* are the fractional flow rates of high-, median-, and low-permeability layers during the agent flooding, respectively, %; *Q* and *Q′* are the fractional flow rates before and after agent flooding, respectively, %; *f_w_* and *f_w_*^′^ are the water cuts before and after agent flooding, respectively, %; *h*, *m*, and *l* represent high-, median-, and low-permeability layers, respectively; *η* is the oil recovery, %; *w* and *p* represent water flooding and agent flooding.

The above three parameters can independently evaluate uniformity, but each has its own limitations. Here, a weighted average method is used to calculate a comprehensive evaluation coefficient based on these three parameters, i.e., DEF, as shown in Equation (8). The weights of the three parameters can be adjusted according to the actual reservoir conditions or on-site requirements.

(8) Ψ=(x×λ+y×ξ+z×θ)

where Ψ is DEF, %; *x*, *y*, *z* represent the contribution rates of profile improvement rate, water cut rise rate, and recovery equilibrium rate to DEF, respectively; the value in this paper is 1/3.

### 4.5. Optimization of Slug Combination Timing

The dynamic change in DEF can be used to guide the switching timing of combination slugs. The three-parallel cores model with 3 median permeability and 3 permeability variation coefficients was used to experiment. HAP and MG flooding was carried out as the scheme of Table 8 (columns A and B), and HPAM flooding with two viscosities was conducted as shown in Table 8 (columns C and D). The DEF of each experiment was calculated based on Equation (8). Define the Production Limit Factor (PLF): the percentage of the oil recovery of a single core in the parallel cores experiment to the ultimate oil recovery of this core. Draw a scatter diagram of DEF and the variation coefficient of PLF of the parallel cores experiment. The DEF value corresponding to the inflection point of the scatter diagram is the optimal time for slug switching. The specific experimental steps and process are the same as in Section 2.2.

### 4.6. Five-Parallel Cores Displacement Experiments

Two five-parallel cores flooding experiments were carried out to verify the effectiveness of the DEF as the basis for the reservoir equilibrium development method. The cores permeability (with a permeability difference of 20) is shown in Table 7, and Figure 13a shows the experimental flow chart. The specific experimental steps are: (1) Preparation work before core flooding. The detailed steps are described in Section 2.2; (2) Five-parallel cores displacement experiment according to Table 10. The switching time of each slug in the scheme was determined according to the DEF curve obtained by real-time calculation; (3) Calculate the water cut, oil recovery, and fractional flow rate of the whole process. The experiment was carried out in a thermostat with a constant temperature of 55 °C, and the injection rate was 1.5 mL/min.

## Figures and Tables

**Table 1:** Key parameters of oil displacement of HAPs and MGs.

Number	Variable	Value	Water Flooding Recovery, %	Agent	Final Recovery, %	EOR, %	Average Profile Improvement Factor, %
1	Coefficient of variation	0.53	23.00	HAP	60.42	37.42	72.00
2			22.99	MG	49.91	26.92	72.31
3		0.76	17.04	HAP	55.01	37.97	59.76
4			15.60	MG	43.73	28.12	60.95
5		0.89	15.31	HAP	48.29	32.99	36.41
6			12.81	MG	39.78	26.97	54.17
7	Median permeability, mD	800	22.80	HAP	61.80	39.00	61.51
8			21.43	MG	46.67	25.24	69.14
9		2000	18.77	HAP	59.01	40.24	61.74
10			18.38	MG	49.28	30.90	63.84
11		5000	17.19	HAP	47.23	30.04	38.19
12			18.77	MG	45.60	26.83	54.26

**Table 2:** Adaptability parameters of full-scale elastic-dispersed fluid reservoirs.

Agent	Coefficient of Variation	Median Permeability, mD	Note
HAP	<0.76	<2000	This adaptability is defined according to experimental parameters and can be used as a design basis for preliminary screening and the combination of systems.
MG	>0.76	>2000	
PPG	/	>10,000 or the maximum reservoir permeability is greater than 10,000	

**Table 3:** The displacement characteristic parameters of different experiments.

Experimental Scheme	EOR of High-Permeability Layer, %	EOR of Median-Permeability layer, %	EOR of Low-Permeability Layer, %	Comprehensive Oil Recovery, %		
				End of Water Flooding	Final	EOR
0.7 PV HPAM	34.79	24.06	18.36	22.98	49.35	26.37
0.7 PV MG	21.37	36.49	17.87	23.81	48.50	24.69
0.7 PV MG + HPAM	19.96	33.20	31.58	24.54	52.53	27.99
0.3 PV MG + 0.4 PV HPAM	25.27	43.23	28.39	25.92	55.67	29.75
0.2 PV HPAM +0.3 PV MG + 0.2 PV HPAM	30.31	40.21	30.84	25.86	59.24	33.37

**Table 4:** Comparison of key oil recovery parameters between conventional conformance control method and full-scale-dispersed fluid method.

Stage		Scheme	
		The Combined Conformance Control Method	The Conventional Conformance Control Method
Final oil recovery	High-permeability layer	67.33	67.75
	Sub-high-permeability layer	63.48	62.86
	Median-permeability layer	64.61	42.02
	Sub-low-permeability layer	37.42	25.73
	Low-permeability layer	30.56	19.91
	Total	54.83	44.65
EOR after HAP flooding	High-permeability layer	0.00	0.00
	Sub-high-permeability layer	5.63	5.54
	Median-permeability layer	31.23	9.11
	Sub-low-permeability layer	25.00	12.50
	Low-permeability layer	23.15	12.69
	Total	17.79	8.10

**Table 5:** Advantages and disadvantages of the four gel-based polymer agents.

Number	Agent	Viscosity	Particle Size	Advantages	Disadvantages	Mainly Used Reservoirs
1	HPAM	Several to hundreds of cP	Approximately 0.2–0.8 μm	Highly fluid, capable of plugging high-permeability layers and penetrating medium- and low-permeability layers, combining the mechanisms of expanding swept volume and improving oil washing efficiency	HPAM is a flexible coil, limiting its plugging effect on high-permeability layers.	Suitable for fracture-free medium- to high-permeability reservoirs
2	HAP	Tens to hundreds of cP	Approximately 0.8–2 μm	Highly fluid, enhancing the plugging effect and limitation of HPAM on high-permeability layers, while also possessing certain interfacial activity.	The matching permeability range is narrow, easily leading to situations where high-permeability layers cannot be plugged and low-permeability layers cannot be injected	Suitable for fracture-free high-permeability reservoirs
3	MG	Approximately 1 to 2 cP	Several to hundreds of μm	Possesses particle elastic deformation and bridging plugging properties, effectively plugging large channels and fractures	The matching permeability range varies depending on particle elasticity, similarly prone to injection failure and inability to plug	Suitable for high-permeability channels or micro-fractured reservoirs
4	PPG	Approximately several to tens of cP	Hundreds of μm	Possesses flexible deformation characteristics, capable of penetrating and plugging larger fractures	Poor injectability	Suitable for fractured reservoirs

**Table 6:** Parameter of the three-parallel cores model.

Number	Variable	Value	Quantity	Low-Permeability Layer, mD	Median-Permeability Layer, mD	High-Permeability Layer, mD
1	Median permeability, mD	800	The permeability ratio is 5	300	800	1500
2		2000		700	2000	3500
3		5000		1500	5000	7500
4	The variation coefficient of permeability	0.53	Median permeability is 2000 mD	800	2000	3500
5		0.76		800	1000	4000
6		0.89		500	1000	4500

**Table 7:** Core parameters of the five-parallel cores model.

Number	Layer	Size, cm	Permeability, mD	Porosity, %	Oil Saturation Rate, %
1	High permeability	2.49 × 30.2	10,000	30.25	78.91
2		2.49 × 30.1	10,000	30.59	77.65
3	Sub-high permeability	2.51 × 30.3	7500	29.87	76.40
4		2.51 × 30.3	7500	29.36	76.22
5	Median permeability	2.50 × 30.1	3000	26.29	75.56
6		2.50 × 30.2	3000	26.29	74.98
7	Sub-low permeability	2.50 × 30.0	1500	25.31	72.25
8		2.50 × 30.2	1500	25.89	73.64
9	Low permeability	2.49 × 30.1	500	25.27	71.20
10		2.49 × 30.0	500	24.98	70.26

**Table 8:** Displacement experimental scheme of reservoir adaptability and slug combination timing optimization.

Number	Variable	Value	Column A	Column B	Column C	Column D
			HAP, mg/L	MG size, μm	HPAM, mPa·s	HPAM, mPa·s
1	Median permeability, mD	800	1000	8.3	45.02	27.60
2		2000	1500	21.0	45.02	27.60
3		5000	2000	21.0	45.02	27.60
4	The variation coefficient of permeability	0.53	1500	21.0	45.02	27.60
5		0.76	1500	21.0	45.02	27.60
6		0.89	1500	21.0	45.02	27.60

**Table 9:** Displacement experimental scheme of slug combination optimization.

Number	Solution	Total Injection Volume, PV	Slug Combination	Displacement Process
1	HPAM, 1000 mg/L	0.7	0.7 PV HPAM	Water flooding to water cut up to 95–Chemical flooding 0.7 PV-Subsequent water
2	MG, 1500 mg/L		0.7 PV MG	
3	HPAM, 1000 mg/L + MG, 1500 mg/L		0.7 PV (HPAM + MG)	
4	HPAM, 1000 mg/L + MG, 1500 mg/L		0.3 PV MG + 0.4 PV HPAM	flooding to the water cut gets to 98%
5	HPAM, 1000 mg/L + MG, 1500 mg/L		0.2 PV HPAM + 0.3 PV MG + 0.2 PV HPAM	

**Table 10:** Five-parallel cores flooding experimental scheme.

Number	Displacement Process	Switching Time	Remark
1	Water flooding to water cut up to 90%—0.7 PV HAP flooding—0.5 PV PPG flooding—0.6 PV HPAM flooding—subsequent water flooding to water cut up to 98%	Switching at the set PV value	Control experiment
2	Water flooding to water cut up to 90%—HAP flooding—HPAM flooding—MG flooding—HPAM flooding—PPG flooding—HPAM flooding—subsequent water flooding to water cut up to 98%	When the DEF < 45% of each slug	Slug combination conformance control method

## Data Availability

The numerical data from Figure 1, Figure 2, Figure 3, Figure 4, Figure 5, Figure 6, Figure 7, Figure 8, Figure 9, Figure 10 and Figure 11 are available as a .zip file in the Supporting Information. This .zip file also includes the main parameters pre- and post-processing, as well as the raw data used for DEF calculation. Additional data that support the findings of this study are available on request from the corresponding author.

## Supplementary Materials

The following supporting information can be downloaded at: https://www.mdpi.com/article/10.3390/gels12060543/s1, Table S1: Matching relationship between HAP and reservoirs with different permeabilities; Table S2: Matching relationship between MG and reservoirs with different permeabilities; Table S3: Matching relationship between PPG and reservoirs with different permeabilities.
