# Supplementary material for: Reservoir Equilibrium Development Method by Combined Conformance Control of Polymer/Gel-Dispersed Fluids
Source: Gels. 2026 Jun 17;12(6):543. doi: 10.3390/gels12060543 (PMC13298229; doi:10.3390/gels12060543)
Supplement: Supplementary file 1 [file gels-12-00543-s001.zip › gels-4306377-supplementary.pdf]

Supplementary Material

Reservoir equilibrium development method by combined profile control of polymer/gels dispersed fluids

Xin Chen [1,*], Jiayi Zhu [1], Yiqiang Li [2], Zheyu Liu [2], Jianbin Liu [1], Houfeng He [1] and Shun Liu [1,*]

[1] College of Petroleum Engineering, Xi'an Shiyou University, Xi'an 710065, China

[2] College of Petroleum Engineering, China University of Petroleum Beijing, Beijing 102249, China
* Correspondence: xchen30@xsyu.edu.cn (X.C.); liushun631@163.com (S.L.)

Table S1 Matching relationship between HAP and reservoirs with different permeabilities

Number	Permeability, mD	Concentration, mg/L	Hydrodynamic characteristic size, μm	Matching relationship
1	500	500	0.65	Difficulty
		1000	1.10	difficulty
		1500	1.80	Hardly
2	1500	500	0.65	smoothly
		1000	1.10	difficulty
		1500	1.80	difficulty
3	3000	500	0.65	smoothly
		1000	1.10	smoothly
		1500	1.80	difficulty
4	4000	1000	1.10	smoothly
		1500	1.80	difficulty
		2000	2.34	difficulty
5	7500	1000	1.10	smoothly
		1500	1.80	smoothly
		2000	2.34	difficulty

Table S2 Matching relationship between MG and reservoirs with different permeabilities

Permeability, mD	Pore-throat size, μm	Particle size, μm	Matching factor	Matching relationship
1000	11.80	4.2	0.36	Smooth through
		8.3	0.70	Optimal matching
		21	1.78	Hardly
2000	16.00	4.2	0.26	Smooth through
		8.3	0.52	Optimal matching
		21	1.31	Strong plugging
3000	19.22	4.2	0.22	Smooth through
		8.3	0.43	Smooth through
		21	1.09	Optimal matching
4000	22.19	4.2	0.19	Smooth through
		8.3	0.37	Smooth through
		21	0.95	Optimal matching
6000	26.67	4.2	0.16	Smooth through
		8.3	0.31	Smooth through
		21	0.79	Weak plugging
		45	1.69	Hardly
7500	29.28	4.2	0.14	Smooth through
		8.3	0.28	Smooth through
		21	0.72	Weak plugging
		40.1	1.37	Strong plugging

Table S3 Matching relationship between PPG and reservoirs with different permeabilities

Permeability, mD	Pore-throat size, μm	Particle size, μm	Matching factor	Matching relationship
6000	26.67	55.5	2.08	Hardly
7500	29.28	55.5	1.90	Hardly
10000	33.22	55.5	1.67	Optimal matching
30000	54.77	55.5	1.01	Optimal matching
